# Endocrine, metabolic, nutritional, and toxic disorders leading to dementia

**DOI:** 10.4103/0972-2327.74247

**Published:** 2010-12

**Authors:** Amitabha Ghosh

**Affiliations:** Department of Neurology and Cognitive Neurology Unit, Apollo Gleneagles Hospitals, Kolkata, India

**Keywords:** Dementia, endocrine, metabolic, nutritional, reversible dementia, toxins

## Abstract

One of the first steps toward the correct diagnosis of dementia is to segregate out the nondegenerative dementias from possible degenerative dementias. Nondegenerative dementias could be due to traumatic, endocrine, metabolic, nutritional, toxic, infective, and immunological causes. They could also be caused by tumors, subdural hematomas, and normal pressure hydrocephalus. Many of the nondegenerative dementias occur at an earlier age and often progress quickly compared to Alzheimer’s disease and other degenerative dementias. Many are treatable or preventable with simple measures. This review aims to give an overview of some of the more important endocrine, metabolic, nutritional, and toxic disorders that may lead to dementia.

## Introduction

In order to correctly diagnose a possible degenerative dementia, it is essential to rule out other nondegenerative causes. Many, but not all, of the nondegenerative causes of dementia are treatable, especially if detected early. A degree of clinical suspicion is, therefore, required. Clues to the diagnosis include an earlier age of onset and a relatively quicker progression compared to Alzheimer’s disease (AD) and other degenerative dementias and a subcortical pattern of cognitive deficit. Diseases such as normal pressure hydrocephalus and subdural hematomas at one end and endocrine, metabolic, infective, immunologic, nutritional, and toxic causes at the other can cause or contribute to the causation of dementia. Toxic causes alone can include environmental toxins, drugs, and irradiation. This review will be restricted to giving an overview of some of the common endocrine, metabolic, and toxic causes, as well as nutritional deficiencies that may lead to dementia.

## Endocrine and Metabolic Causes

Both clinical hypothyroidism and hyperthyroidism have long been linked with reversible cognitive impairment in patients.[1,2] Thyroid function tests are also recognized for the work-up for patients with dementia. Recent studies have suggested an association between cognitive impairment and high, as well as low thyroid stimulating hormone (TSH) levels, even when these values are within normal range.[3,4] Others, however, have failed to reproduce these findings.[5,6] The question regarding the role of thyroid hormone dysfunction in nonreversible dementias such as AD is still open. A recent carefully controlled study of 1864 cognitively intact, clinically euthyroid participants in the Framingham study found that both high and low TSH levels were associated with increased risk of developing incident AD in women, but not in men.[7] The exact mechanisms are still unclear. Low thyroid hormone levels in the central nervous system (CNS) may directly increase amyloid precursor protein (APP) expression, thereby increasing A-beta production, leading in time to AD. A direct effect of thyroxine depletion on cholinergic neurons has also been suggested. Conversely, chronically high thyroid hormone levels have been associated with AD, probably through progressive acetylcholine depletion, thereby giving rise to cognitive problems linked with the cholinergic deficit.[7] A community-based study in the elderly showed a threefold increase in dementia and AD in patients with baseline subclinical hyperthyroidism.[8] AD, by itself, can cause an undersecretion of thyrotrophin releasing hormone (TRH) from the hypothalamus. The low TRH level may, in turn, act in two ways. It may lead to a reduced production of TSH from the pituitary, leading to low thyroxine levels. The low TRH may also lead to increased phosphorylation of tau proteins which is the pathological manifestation of AD.[9] However, other researchers, looking for early imaging evidence for the risk of developing AD, failed to find a significant role of thyroid dysfunction.[10]

Of note, a small study of 129 patients with dementia from India did not observe patients with thyroid hormone disorders.[11]

Although not directly caused by an altered thyroid hormone level, Hashimoto encephalopathy[12] merits mention. This is a steroid responsive encephalopathy associated with autoimmune thyroiditis. Antithyroid antibodies to thyroid peroxidise or thyroglobulin are frequently elevated. Presentation may be nonspecific and variable, but in some patients it may take the form of a subacute cognitive impairment. Associated myoclonus could lead to a mistaken diagnosis of Creutzfeldt–Jacob disease (CJD) or diffuse Lewy body disease, both of which should be considered in the differential diagnosis. Magnetic resonance imaging (MRI) scans of the brain may show nonspecific white matter changes and cerebrospinal fluid (CSF) protein may be raised. However, these results may be normal or nearly so, and a high index of clinical suspicion is required in such situations.[13]

Cognitive impairment, dementia, and psychoses have been described in patients with chronic hypocalcemia, hypoparathyroidism, and hypercortisolism. Chronic endogenous secretion as well as exogenous administration of steroids may lead to cognitive impairment largely by an adverse effect upon hippocampal function. Patients receiving chronic corticosteroid therapy have been shown to have smaller hippocampal volumes and declarative memory deficits.[14] It is generally believed that these effects on memory and on hippocampal volumes tend to occur more when higher doses of steroids are used. Similar association between reduced hippocampal volume, memory dysfunction, and elevated cortisol levels have been reported in patients with Cushing’s syndrome.[15]

Repeated episodes of hypoglycemia can give rise to cognitive impairment and dementia. In a recent study of older patients with type 2 diabetes mellitus, a history of recurrent severe hypoglycemic episodes was associated with a greater risk of dementia.[16] It is possible that recurrent minor hypoglycemic episodes during the course of diabetes mellitus may not be as detrimental.

Postischemic–hypoxic encephalopathy, after recovery from cardiac arrest for example, can lead to amnesia or more profound dementia. The degree and persistence of cognitive loss depend upon the duration of ischemic–hypoxic injury to susceptible cortical neurons in the hippocampus and elsewhere. Typical areas that are susceptible to such insults include the CA1 and CA3 regions of the hippocampus, layers 3, 5, and 6 of the neocortex, and the Purkinje cells of the cerebellum.[17]

Carbon monoxide (CO) poisoning, an example of nonischemic hypoxia, can lead to a delayed onset cognitive decline beginning days to several weeks after apparent recovery from the initial insult. Tissue hypoxia in patients with CO intoxication is caused by the strong affinity of CO for hemoglobin, leading to the displacement of oxygen from the binding sites. CO also affects mitochondrial function and may additionally have a direct neurotoxic effect. Cognitive decline, personality changes, urinary or faecal incontinence, parkinsonism, and even mutism may occur.[18,19] Prognosis is relatively good with the majority of victims recovering within 1 year.

Hepatic or portal systemic encephalopathy, even when minimal, can alter cognitive performance.[20] Cirrhotic patients without overt features of encephalopathy have repeatedly been shown to underperform on neuropsychological tests.[21–23] Hyperammonemia may be central to its causation and the clinical state of the patient correlates well with arterial ammonia levels. Endogenous benzodiazepine ligands may also contribute. Electroencephalography (EEG) may show slow waves or triphasic waves. Increased signal intensity in the globus pallidus on T1-weighted MR images of the brain is typical. Much of the cognitive dysfunction may improve with treatment. Occasionally, patients with cirrhosis develop a progressive cognitive and extrapyramidal syndrome similar to Wilson’s disease called acquired hepatocerebral degeneration.[24] This typically occurs after repeated episodes of liver failure. Irreversible dementia may occur. Medical treatment is unsatisfactory, but liver transplantation may be helpful in selected cases.[25,26]

Cognitive impairment is common and may potentially occur in up to 80% of patients with chronic kidney disease (CKD). The prevalence of cognitive impairment increases with the severity of disease. While acute cognitive impairment can be caused by electrolyte disturbances, cerebral hypoperfusion from acute fluid shifts during dialysis, malignant hypertension, and the now uncommon “dialysis disequilibrium syndrome”, chronic cognitive dysfunction and dementia are also significantly increased independent of other risk factors.[27] Memory and executive functions are most commonly affected.[28] Although cerebrovascular disease and, in particular, silent cerebral infarctions are common in CKD, the direct link between vascular risk factors and cognitive impairment seen in patients with CKD has not been convincingly established.[27] Newer evidence points toward a direct role of inflammatory mediators in producing cognitive dysfunction.[29] The now uncommon “dialysis dementia” was caused by the use of aluminum-rich dialysate fluid used earlier. Its incidence has dropped drastically with the removal of aluminum from the dialysate fluid. The use of non-aluminum phosphorus binders has also helped. Recent research has implicated hyperparathyroidism and anemia in the causation of dementia in CKD.[29] Considerable improvement in cognitive performance and especially in memory occurs after renal transplantation.[30]

## Adult-onset inherited disorders of metabolism causing dementia

Metachromatic leukodystrophy is an autosomal recessive inherited disorder of metabolism, most commonly caused by a deficiency of the enzyme arylsulfatase A. Abnormal accumulation of sulfatides in the form of metachromatic granules in the oligodendrocytes and Schwann cells leads to both central and peripheral demyelination. A slowly progressive dementia and behavioral dysfunction including personality changes and psychoses may be early manifestations of the adult form of the disease. MR imaging of the brain typically shows diffuse, symmetrical, confluent areas of increased intensity in the white matter on T2-weighted and FLAIR sequences, but multiple nonenhancing plaques may also occur. U-fibers are typically spared. Cerebrospinal protein levels may be markedly raised. Arylsulfatase A activity in the peripheral leucocytes is reduced, and sulfatides are seen to accumulate in the urinary sediment. Bone marrow transplantation may be helpful to some extent.

Adrenoleukodystrophy (ALD) is an X-linked recessive disorder of peroxismal membrane-located protein that impairs transport of very long chain fatty acids (VLCFA) into peroxisomes, thereby impairing peroxismal β-oxidation of VLCFA.[31] The VLCFA then accumulates in oligodendrocytes, Schwann cells, and adrenal cortical cells, resulting in destruction of myelin in the central and peripheral nervous system, as well as causing Addison’s disease. Psychiatric symptoms and cognitive decline leading to dementia have been described in adrenomyeloneuropathy (AMN), and the adult cerebral forms of the disease. MRI scan shows confluent white matter hyperintensities on T2-weighted and FLAIR images. These are typically seen over the parieto-occipital regions and involve the splenium, although frontally predominant lesions may also occur. Enhancement of the lesional margins indicates disease progression. MR spectroscopy (MRS) shows reduced *N*-acetyl aspartate activity and an increase in choline peaks sometimes with a milder increase in the lactate peak. Treatment of established neurological disease is currently of limited benefit and rests on dietary measures to reduce VLCFA levels, bone marrow transplantation and hematopoietic stem cell therapy (HSCT).[32]

Adult polyglucosan body disease[33] is a slowly progressive rare leukodystrophy characterized by deposition of periodic acid-Schiff positive polyglucosan bodies in the central and peripheral nervous system. A mild dementia may occur in addition to progressive bladder dysfunction, mixed upper and lower motor neuron involvement, and a distal sensory loss, especially in the leg. MRI of the brain may show extensive white matter changes together with diffuse atrophy.[34]

Cerebrotendinous xanthomatosis is caused by a mutation in a gene called CYP27A1, which produces the enzyme, sterol 27-hydroxylase. Adult forms can present with a variety of neurological symptoms including psychiatric disorders and cognitive impairment leading to dementia. Timely treatment with chenodeoxycholic acid may halt, slow, and in some cases reverse the course of the disease.[35]

Kuf’s disease or the adult form of neuronal ceroid lipofuscinosis can present with behavioral symptoms followed by progressive dementia together with motor symptoms and epilepsy. Pigmentary retinal degeneration and blindness are typically absent in the adult variety. Skin biopsy may assist in the diagnosis. Bone marrow transplantation has been tried for treatment.

Cognitive impairment and dementia may be seen in a minority of patients with advanced Wilson’s disease. It is typically mild and may even reverse with treatment.[36–38] Mitochondrial disorders including mitochondrial encephalomyopathy, lactic acidosis, and stroke-like episodes (MELAS) and Leigh’s disease may present with cognitive dysfunction. Patients with Fahr’s disease, or basal ganglia calcification, may present with dementia or may progress to dementia later during the course of their illness.[39–41] The dementia may not be typical of any degenerative dementia, but may have mixed features instead. Neuropathological findings are varied and could include frontotemporal atrophy with white matter demyelination and fibrous gliosis seen in the atrophied areas, together with widespread neocortical neurofibrillary tangles but no neuritic plaques. Neuronal loss in the nucleus basalis of Meynert and calcareous deposits are also seen.[42]

## Nutritional Deficiencies

Memory loss may be seen in patients with the Wernicke–Korsakoff syndrome. This occurs as a result of thiamine deficiency that may be seen in chronic alcoholics or in malnourished patients. Typically, Wernicke’s encephalopathy occurs first, with nystagmus, ophthalmoplegia and ataxia, and an acute confusional state. Untreated patients develop progressive drowsiness, coma, and even death. Early treatment with thiamine produces fast and dramatic improvement. The Korsakoff amnesia usually sets in as the Wernicke’s encephalopathy improves, although other presentations are also recognized. Cognitive loss takes the form retrograde and anterograde amnesia that occur far in excess of other cognitive symptoms. Lesions in the medial thalamus and its connections are believed to be responsible for the memory deficit.[43] The mamillary bodies, periaqueductal gray matter, cerebellar vermis, and other areas are also involved. Confabulation is common, and there is marked difficulty in putting past events in the correct temporal sequence. This results from the involvement of the frontal fibers. Once established, the amnesia of Korsakoff’s syndrome is difficult to eradicate in the majority of patients in spite of adequate doses of thiamine. An early diagnosis of the Wernicke’s encephalopathy, largely based on clinical suspicion, is therefore essential.

Pellagra, caused by niacin deficiency, is characterized by the classical triad of dermatitis, diarrhoea, and dementia. Polyneuropathy and dorsal column involvement in the spinal cord also occur. Malnutrition, alcoholism, and anorexia nervosa, among other causes, can give rise the niacin deficiency. The symptoms reverse rapidly with niacin replacement.

The role of vitamin B-12/folate levels in the development of dementia is more controversial. Vitamin B-12 deficiency may occur in pernicious anemia, celiac disease, gastric and ileal resections, blind loop syndrome, fish tapeworm infestation, and in strict vegans. Subacute combined degeneration is its commonest neurological manifestation. Neuropsychiatric symptoms are common, but a progressive dementia caused by vitamin B-12 deficiency alone is less frequent. It has been argued that patients who are deficient in vitamin B-12 or folate may have a higher risk of cognitive impairment in the presence of a high serum homocysteine level.[44] Other studies have demonstrated an independent association of low vitamin B-12 concentrations with cognitive decline. In a recent longitudinal cohort study between 1993 and 2003, cognitive function was studied against serum concentrations of vitamin B-12, holotranscobalamin (holoTC; the biologically active fraction of vitamin B-12), total homocysteine (tHCY), methylmalonic acid (MMA), and folate. Low serum concentrations of holoTC and high MMA levels (both of which are indicators of low vitamin B-12 status) were independently and significantly associated with a more rapid cognitive decline over the 10-year study period.[45] In a study of 129 patients with dementia in India, 5 patients were found to have vitamin B-12 deficiency. The authors mention that vitamin B-12 level estimation in this study was not done in all patients but only as deemed necessary. Of note, only two out of the five patients with vitamin B-12 deficiency had been clinically suspected. All the five patients improved with treatment.[11] Neurological symptoms of vitamin B-12 deficiency need to be treated with parenteral supplementation as early as possible for the best results.

## Toxins

### Alcohol

Some of the nutritional deficiency states causing dementia and cognitive impairment in chronic alcoholics have already been described. Here, we mention two other alcohol-related dementias, namely the Marchiafava–Bignami disease and the so-called “alcoholic dementia”. Marchiafava-Bignami disease is seen mainly in male chronic severe alcoholics. Varied neurological presentations have been described, including a rapidly progressive dementia in some patients. Cognitive impairment may take the form of a frontal lobe syndrome. Pathologically, the middle part of corpus callosum is characteristically affected but anterior and posterior parts of the corpus callosum, the anterior and posterior commisures, and some other parts of the brain may also be involved. Radiological changes corresponding to the lesions may be evident on CT and MR scans of the brain. While most patients have a progressive illness which may lead to death, some stabilize, while others may even show some improvement with adequate nutrition and thiamine replacement.

Alcoholic dementia is a vaguely defined entity and probably consists of Wernicke–Korsakoff syndrome, Marchiafava–Bignami disease, hepatic encephalopathy, head injury, subdural hematoma, normal pressure hydrocephalus, vascular cognitive impairment, or AD, alone or in various combinations.

### Heavy metals

#### Mercury

Both organic and inorganic mercury has been linked to the pathological and neurochemical changes that may be seen in AD.[46,47] In humans, one of the likely sources of organic mercury such as methyl mercury is the consumption of contaminated fish. Dental amalgams are a common source of inorganic mercury. Chronic inhalation of mercury vapor historically occurred to persons employed in the felt hat industry. A resultant encephalopathy, characterized by tremor and cognitive and behavioral decline is prominent in this setting (hence the term “mad hatter”). Penicillamine and dimercaptosuccinic acid (succimer) are useful for the treatment of chronic mercury intoxication, while BAL, which increases the concentration of mercury in the brain, is not.

#### Arsenic

Chronic arsenic encephalopathy may affect cognition and personality and may precipitate psychotic symptoms such as delusions and hallucinations. Groundwater contamination is a major source of chronic arsenic toxicity in parts of West Bengal and Bangladesh.[48,49] Arsenic inhibits mitochondrial function and interferes with oxidative metabolism in neurons.[50] Some improvement of symptoms may follow removal of the exposure.

#### Lead

Cognitive decline caused by chronic exposure to lead may occur long after cessation of the exposure. An accelerated longitudinal decline in cognitive function has been observed in adult workers who are no longer exposed to lead in their workplace.[51] These patients have been shown to have lower brain volumes and an increase in signal intensity on T2-weighted MR images of the brain.

### Toluene

Chronic toluene exposure, for example, following chronic recreational use of glue, gasoline and paint, may lead to cognitive and behavioral problems. Toluene is highly lipid soluble, crosses the blood–brain barrier easily and may damage myelin and cause neuronal cell death.

### Lithium

Lithium is currently being investigated for its neuroprotective effect against dementia especially in patients with bipolar depression, and as a disease modifying agent in AD.[52,53] Nevertheless, a subacute dementia with myoclonus and periodic sharp waves on EEG, mimicking Creutzfeldt–Jacob disease may occur with chronic lithium toxicity.[54–56] The symptoms subside after lithium is stopped. Awareness about this toxicity is, therefore, helpful for physicians who regularly treat patients receiving lithium.[57] A similar picture can also be caused by *bismuth* toxicity from chronic high dose abuse. Recovery follows early discontinuation.[58]

### Drugs

Chronic intake of benzodiazepines and psychotropic drugs may lead to or worsen an underlying cognitive impairment. Long-term antiepileptic therapy with phenobarbitone, phenytoin, carbamazepine, or sodium valproate may have a similar effect. Sodium valproate-induced hyperammonemic encephalopathy (VHE), also known as valproate-induced encephalopathy (VIE) is an under-recognized condition that presents with varying degrees of cognitive and behavioral dysfunction.[59–61] Progressive dementia may be seen in elderly patients. No prior hepatic dysfunction need to be present. A second antiepileptic drug, particularly topiramate, may act as a precipitating factor. Withdrawal of valproate therapy and administration of l-carnitine decrease ammonia levels and reverse the symptoms.

The newer antiepileptic drugs, by themselves, appear to have less cognitive side-effects.

### Radiation

A late-delayed encephalopathy may occur, several months or years after whole brain irradiation, for example for metastatic tumor in adults. Progressive memory loss, sometimes with ataxia and urinary incontinence may be seen.[62] Cognitive impairment has also been noted in patients receiving prophylactic cranial radiation for small-cell lung cancer,[63] while it has been variably reported in patients after the treatment of focal brain tumors.[64,65] Cognitive loss leading to learning disabilities can also be seen in children receiving prophylactic whole brain irradiation for acute lymphoblastic leukemia. Manifestations of the late delayed effects of radiotherapy on brain function are related to the patient’s age, total dose of irradiation, fraction sizes, and timing of chemotherapy. MRI of the brain in patients with cognitive loss may be normal or, in patients receiving higher doses of radiation, may show cerebral atrophy with ventriculomegaly. Periventricular hyperintensities on T2-weighted or FLAIR sequences may also be seen. Pathologically, spongiform changes are seen in the white matter. Treatment is limited although shunting of CSF may benefit some of the patients presenting with a picture typical of normal pressure hydrocephalus.

### And finally… the enigma of cyad

The island of Guam, in the Western Pacific, is inhabited by the Chamorro people, a native to this area. Around 60 years ago, the Chamorro were discovered to have an unusually high incidence of neurodegenerative disorders and in particular a disease characterized by the combination of anterior horn cell disease, parkinsonism and dementia (ALS/PDC).[66,67] Both the parkinsonism and dementia in this complex could be severe and were associated with abundant Alzheimer-like tau-positive neurofibrillary tangles, but without the plaques. Why these diseases were common here remains an unresolved mystery, but genetic and environmental factors have both been implicated. There is evidence to suggest that a dietary toxin consumed by the Chamorro might have played a role. The Chamorro consumed cyad (also called cycad), the seed of the false sago palm which, like the Chamorro, is native to the Western Pacific. It is also used for a topical medicine. Cyad may be eaten in two ways. Traditional Guamanian food included cyad flour. The Chamorro also ate fruit bats for special meals. Fruit bats consume large amounts of cyad seeds, which then bioaccumulate in high concentrations in their tissues.[68] Consuming these bats transfers heavy doses of the neurotoxins present in the cyad into the person. It is interesting that over the last several decades, with changing eating habits, many among the Chamorro people have moved away from eating cyad flour and fruit bats. This coincides with a dramatic reduction in the number of ALS reported over this period suggesting the role of a modifiable environmental risk factor. However, the incidence of PDC has not declined as much, with an unchanged clinical picture apart from an onset later in life.[69] Furthermore, a late-life dementia without early parkinsonism has been described in the Chamorro elderly. Although clinically akin to AD, the neuropathological and genetic findings bear a closer relation to ALS/PDC.[70]
